# Exposure to visual cues of pathogen contagion changes preferences for masculinity and symmetry in opposite-sex faces

**DOI:** 10.1098/rspb.2010.1925

**Published:** 2010-12-01

**Authors:** Anthony C. Little, Lisa M. DeBruine, Benedict C. Jones

**Affiliations:** 1 Department of Psychology, University of Stirling, Stirling, UK; 2 School of Psychology, University of Aberdeen, Aberdeen, UK

**Keywords:** sexual dimorphism, asymmetry, attractiveness, pathogens, disease, variation

## Abstract

Evolutionary approaches to human attractiveness have documented several traits that are proposed to be attractive across individuals and cultures, although both cross-individual and cross-cultural variations are also often found. Previous studies show that parasite prevalence and mortality/health are related to cultural variation in preferences for attractive traits. Visual experience of pathogen cues may mediate such variable preferences. Here we showed individuals slideshows of images with cues to low and high pathogen prevalence and measured their visual preferences for face traits. We found that both men and women moderated their preferences for facial masculinity and symmetry according to recent experience of visual cues to environmental pathogens. Change in preferences was seen mainly for opposite-sex faces, with women preferring more masculine and more symmetric male faces and men preferring more feminine and more symmetric female faces after exposure to pathogen cues than when not exposed to such cues. Cues to environmental pathogens had no significant effects on preferences for same-sex faces. These data complement studies of cross-cultural differences in preferences by suggesting a mechanism for variation in mate preferences. Similar visual experience could lead to within-cultural agreement and differing visual experience could lead to cross-cultural variation. Overall, our data demonstrate that preferences can be strategically flexible according to recent visual experience with pathogen cues. Given that cues to pathogens may signal an increase in contagion/mortality risk, it may be adaptive to shift visual preferences in favour of proposed good-gene markers in environments where such cues are more evident.

## 1. Introduction

Evolutionary approaches to human attractiveness have documented several traits that are proposed to be attractive across individuals and cultures, potentially reflecting species-wide ‘universal’ preferences. These include preferences for facial traits such as symmetry and sexually dimorphic cues [1]. Several researchers have proposed that symmetry and sexually dimorphic traits (masculine appearance in men and feminine appearance in women) in human faces may be cues to heritable fitness benefits and therefore relate to attractiveness (e.g. [1]).

Symmetry has long been proposed to be associated with male and female genotypic quality (e.g. [2]). Deviations from perfect symmetry can be considered a reflection of imperfect development. It has been suggested that only high-quality individuals can maintain symmetric development under environmental and genetic stress and therefore symmetry can serve as an indicator of phenotypic quality as well as genotypic quality (e.g. the ability to resist disease; see [3] for review). Consistent with this proposal, more asymmetric men and women have been found to report more health problems [4], although not all studies have found a relationship between symmetry and health [5]. Both studies of real faces [6–9] and recent studies manipulating symmetry [10–13] provide evidence that symmetry is indeed found attractive. While subtle facial asymmetries significantly impact on attractiveness, the relationship is not strong (e.g. [1]).

Masculine facial traits (large jaws, prominent brows) in males are thought to be testosterone-dependent and, consequently, may represent an honest immunocompetence handicap signalling quality [14]. Indeed, masculine-faced men do report having lower incidence of disease [4] and better medical health [15]. Although there is some evidence that masculine male faces are found attractive (e.g. [6,16,17]), several studies have shown that feminine faces and faces of low dominance are also attractive [18–20]. This suggests that judgements of male facial attractiveness may depend on more than just cues to ‘good genes’ for immunocompetence (e.g. [21]). In women, facial attractiveness correlates with body attractiveness [22] and oestrogen-dependent characteristics of the female body correlate with health and reproductive fitness [23]. Increasing the sexual dimorphism of female faces should therefore enhance attractiveness as oestrogen also affects facial growth [24]. Indeed, there is considerable evidence that feminine female faces and faces of women with high oestrogen are considered attractive (e.g. [25]). Studies measuring facial features from photographs of women [6,26,27] and studies manipulating feminine traits in both real [28] and composite [20] faces all indicate that femininity increase the attractiveness of female faces.

Overall, there is support for the notion that sexual dimorphism and symmetry in faces advertise some aspects of quality and are preferred. Indeed, symmetry and sexual dimorphism are correlated in male and female faces [29], and preferences for these characteristics in opposite-sex faces are positively correlated [30]. Importantly, systematic variation is seen in preferences for these facial cues. Previous studies have examined preference for masculine and feminine traits in faces showing that, at least in women, preferences can change between individuals according to condition (as measured by self-perceived and rated attractiveness [10,31]) and partnership status [20], within individuals according to hormonal fluctuations (e.g. across the menstrual cycle [32–34]) and within individuals according to the temporal context of relationship (short- versus long-term [21]). Women prefer relatively more masculine-faced men when they think themselves attractive, when they already have a partner, at peak fertility in the menstrual cycle and when rating for short-term relationships. These findings have been interpreted as consistent with the idea that masculinity in male faces is associated with good genes (i.e. they advertise genetic quality [1]), as these are conditions under which we might expect women to be most attentive to potentially heritable genetic benefits. While less studied, similar results indicating individual differences in preference have been seen for men judging female faces [35–37] and for men and women judging symmetry [10,33,38].

The reason for individual variation in attraction to masculinity and symmetry may lie in a trade-off between genetic quality and investment [21,39]. High-quality individuals may invest less in each partner (and offspring) or be more likely to cheat on/desert partners. High-quality individuals may not make ideal long-term partners in a species such as humans with extended parental investment [40,41]. For example, high-testosterone men are less likely to marry, more likely to divorce and have more marital problems than lower-testosterone men [42], and masculine-faced men are also perceived as poor-quality parents [20]. Previous studies have mainly focused on individual differences based on factors intrinsic to the choosing individuals (e.g. physical attractiveness), but we may also expect variation according to extrinsic ecological conditions that influence the relative value of high parental investment versus good-gene/high-fertility benefits from partners. For example, the degree of harshness and pathogen stress in the environment an individual inhabits might influence the trade-off between a high-investing partner and one with good genes, as it is known to influence reproductively important outcomes and processes such as the age of childbearing, sperm concentration and quality, coital frequency, menstrual and hormonal cyclicity, fertility, birth rates, and breast milk supply [43–46].

Under conditions of low resources, a preference for an investing partner via a low-mating-effort/high-parental-investment strategy may be adaptive, whereas under conditions of relatively high resources, a choice for ‘good genes’ via a high-mating-effort/low-parental-investment strategy may be a better option [47,48]. For example, in a ‘harsh’ environment that has low resource availability, having a stable partner may be of increased importance, particularly for women during pregnancy, as the resources to raise a child may be scarce or difficult to acquire. Thus, two parents to provide the resources necessary for offspring survival and eventual reproduction may be better than one. Likewise, safe environments that have high resource availability may favour the choice of good genes, as an individual can acquire the resources they need themselves. Essentially, there may be little gain in terms of offspring survival/reproduction by the additional effort of a second parent.

Alternatively, in a harsh environment where high extrinsic mortality is greater, such as in high-pathogen-risk populations, the probability of offspring survival and eventual reproduction decreases. Consequently, there may be few benefits to attracting an attentive/investing partner, because individuals may maximize their reproductive output by focusing on acquiring good genes for their offspring to be able to thrive in the hostile environment (e.g. [49,50]). However, in an environment with low mortality rates, the probability of offspring survival and eventual reproduction is greater, and thus choosing an investing partner aids in channelling those resources to the care of relatively few, competitive offspring [49,50].

Recent cross-cultural studies have examined these issues by testing for variation in preferences across cultures varying in environmental stressors. Penton-Voak *et al*. [51] found stronger preferences for male masculinity in Jamaicans than in the UK and Japan. They suggested that a higher pathogen prevalence may result in increased preferences for masculinity in male faces, as it has been shown that pathogen load is positively related to the importance of physical attractiveness in mate choice across different cultures [52] and that masculinity is preferred more under conditions where women may acquire genetic benefits to offspring [21,32]. The Hadza, a tribe of African hunter–gatherers, have been found to exhibit stronger preferences for facial symmetry than do participants in the UK [38]. Following the same logic as Penton-Voak *et al*. [51], a difference in pathogen load between samples may also explain increased preferences for symmetry in the Hadza because individuals close to the equator have higher pathogen loads [53] and outdoor living is likely to increase exposure to pathogens. A more recent study examined a larger cross-cultural sample of 30 countries, calculating both the average female preference for male facial masculinity and a composite health index derived from World Health Organization statistics [54]. This study found that poorer health (i.e. higher mortality and incidence of disease) was related to stronger female preferences for male masculinity [54].

Consistent with these studies, DeBruine *et al*. [55] also demonstrated a correlation between women's preference for masculine male face shape and sensitivity to pathogen disgust. Women who were more disgusted by pathogens showed stronger preferences for masculine male faces, while no such relationship was found for moral or sexual disgust. This study suggests that individual differences in sensitivity to pathogens may explain some variation in women's masculinity preferences within a culture.

Results from all of these studies indicate that prevalence of and sensitivity to pathogens are potentially important determinants of mate preferences, but these studies are correlational and do not address how such associations arise. Indeed, a re-analysis of the data presented in DeBruine *et al*. [54] suggested that factors associated with male–male competition (e.g. homicide rates) might also be associated with variation in preferences for masculinity in women across cultures [56]. The current study tested for a mechanism by which such variable preferences may come about by examining the effect of exposure to visual cues to pathogens on symmetry and masculinity/femininity preferences in both men and women. Following demonstrations that preferences for cues of genetic quality are higher in cultures with higher pathogen stress and among women who are particularly sensitive to pathogens, we hypothesized that exposure to visual cues to pathogens would increase women's preference for masculine- and symmetric-faced men, and men's preference for feminine- and symmetric-faced women.

## 2. Methods

### (a) Participants

One hundred and twenty-four women (aged between 17 and 45 years, mean = 24.8, s.d. = 6.6) and 117 men (aged between 17 and 45 years, mean = 26.9, s.d. = 7.4) took part in the study. Participants were selected for being older than 16 and less than 46 years of age and reporting to be heterosexual. Participants were recruited for the study online via a research-based website (www.alittlelab.com) and the study was conducted online. Previous research has shown that systematic variation in men's and women's face preferences observed in online studies is very similar to that seen in laboratory studies (e.g. [33,37,57]).

### (b) Stimuli

All images were photographs of white individuals (aged between 18 and 25) without spectacles or obvious facial hair. Photographs were taken under standardized lighting conditions and with participants posing with a neutral expression. To equate size, all images were aligned to standardize the position of the pupils in the image. As we are testing whether exposure to pathogen-related stimuli can shift preferences, it is important that preferences for sexually dimorphic shape and symmetry are not at ceiling. Therefore, our manipulations are purposefully subtle.

### (c) Sexually dimorphic shape

**Figure 1.**
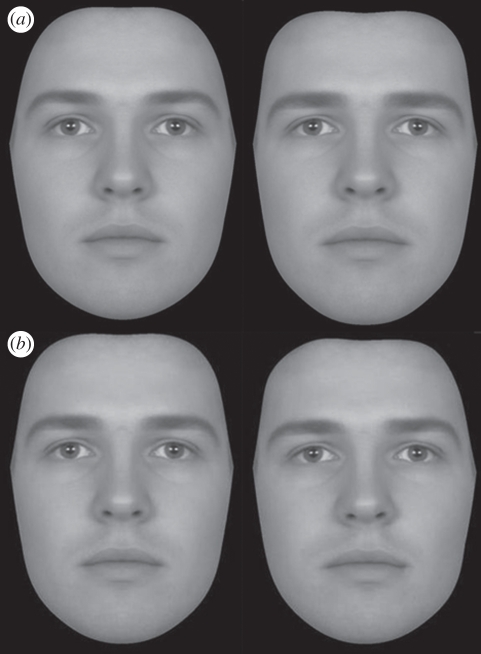
(*a*) Feminized (left) and masculinized (right) male faces. (*b*) Symmetric (left) and asymmetric (right) male faces.

To measure preferences for sexually dimorphic features, we used pairs of composite face images. The pairs comprised one masculinized and one feminized version of the same face (figure 1). Images were manufactured from 50 young adult Caucasian male and 50 female photographs. Composite images, composed of multiple images of different individuals, were used as base faces (10 male and 10 female composite images each made of five individual images). The composite images were made by creating an average image made up of five randomly assigned individual facial photographs [19] (this technique has been used to create composite images in previous studies; see [58,59]). Faces were transformed on a sexual dimorphism dimension using the linear difference between a composite of all 50 male faces and a composite of all 50 female faces (following the technique reported by Perrett *et al*. [20]). Transforms represented ±50 per cent of the difference between these two composites (100% would represent the complete transform, so starting from a female face and transforming by 100% towards male would make the face into a perceptually male shape). This meant that the face was transformed along the sexual dimorphism axis, either increasing masculinity or increasing femininity, and that the face retained its identity and perceived sex (i.e. the faces remained male or female in appearance). All composite images were made perfectly symmetric prior to transform so that transforms did not manipulate symmetry. Final images were 20 feminine/masculine pairs (10 female, 10 male).

### (d) Symmetry

To measure preferences for symmetry, we used pairs of composite face images. The pairs comprised one symmetric and one asymmetric version of the same face (figure 1). Composite images were the same as outlined above (five male and five female images). Images were made perfectly symmetric and then a transform applied. The transform applied was different for each image, representing the difference between an original image and its symmetric counterpart. In this way, the transform then applied the asymmetry apparent in an original individual image. A similar technique, though not using composites, has been used in previous studies [33]. The transform then created two images, one symmetric and one asymmetric, for each base face. Images were then masked on the outline of the face so that hair and clothing cues were not visible in the image. Figure 1 shows an example of transformed faces made using these methods. Final images were 10 symmetric/asymmetric pairs (five females, five males).

### (e) Cues to high/low pathogen incidence

Images of objects holding a potential disease threat were taken from a published study examining cues to pathogens and how disgusting they are seen as [60]. Images were pairs, for example, depicting a white cloth with either a stain resembling bodily fluid (high pathogen) or a stain of blue liquid (low pathogen). In their study, seven out of eight of the high-pathogen stimuli were reported as significantly more disgusting than the paired image, designated here as low-pathogen stimuli, and this same pattern of results was found across many cultures [60]. For our study, the seven image pairs that were consistently seen as differing in disgust perception were extracted from a high-quality PDF of the stimuli. The eighth stimulus pair presented in the study was not used, as it was not consistently seen as more or less disgusting across cultures [60].

### (f) Procedure

Participants were administered a short questionnaire assessing age, sex and sexual orientation, followed by the main test. The main test consisted of three parts: an initial test that assessed participants' preferences for symmetry and sexual dimorphism in own- and opposite-sex faces (the pre-exposure test), a slideshow of either high- or low-pathogen images (the exposure phase), and a post-exposure test that was identical to the pre-exposure test. Participants were told ‘In this study you will see faces to rate for attractiveness. You will also see a slideshow of things you may or may not find disgusting and then be asked to judge the images again’. No other information about why the slideshow was presented was provided.

In the pre-exposure test, the 20 pairs of masculine and feminine faces and 10 pairs of symmetric and asymmetric faces were shown with both order and side of presentation randomized. Participants were asked to choose the face from the pair that they found most attractive. Clicking a button moved participants on to the next face trial. Image order and side of presentation were randomized. The methods used to assess men's and women's preferences for symmetry and sexual dimorphism have been used in previous studies [14,30]. In the exposure phase, participants saw a slideshow of seven images repeated three times (for a total of 21 images) with either cues to high or low incidence of environmental pathogens. Images were presented for 3 s each (for a total of 63 s of exposure) with instructions: ‘Please try and look at these images carefully’. Image order was randomized. The post-exposure test followed and was identical to the pre-exposure test.

## 3. Results

For each participant, we calculated the percentage of faces with increased sexual dimorphism (i.e. feminine female and masculine male faces) chosen out of the 10 pairs of male and female faces and also the percentage of symmetric faces chosen out of the five pairs of male and female faces. This was done separately for the pre- and post-exposure tests, giving four scores on pre-exposure and four scores on post-exposure.

One-sample *t*-tests against chance (50%) revealed that women significantly preferred feminine female faces, masculine male faces and symmetric female and male faces in both the pre-exposure and post-exposure tests. Men significantly preferred feminine female and feminine male faces, and symmetric female and male faces, in both the pre-exposure and post-exposure tests (see table 1 for data; all *p* < 0.001).

**Table 1.** Means and standard deviations for preferences for sexually dimorphic shape (masculinity for women and femininity for men) and symmetry in male and female faces for male (*n* = 117) and female (*n* = 124) participants.

	female		male	
	mean (s.d.)	*t*	mean (s.d.)	*t*
*sexually dimorphic shape*				
female faces				
pre-exposure	68% (23.4)	8.54*	68.9% (24.6)	7.87*
post-exposure	66.5% (25.9)	7.11*	69.8% (24.2)	8.87*
male faces				
pre-exposure	58.6% (27.1)	3.52*	40.6% (26.5)	3.84*
post-exposure	60% (26.7)	4.13*	41.4% (26.3)	3.54*
*symmetry*				
female faces				
pre-exposure	72.7% (23.4)	10.81*	73.7% (25.8)	9.92*
post-exposure	68.4% (27.0)	7.58*	75.2% (24.9)	10.97*
male faces				
pre-exposure	62.7% (25.8)	5.50*	71.3% (24.0)	9.60*
post-exposure	64.4% (26.5)	6.04*	72% (23.9)	9.93*

*Denotes *p* < 0.001.

To examine the change in preference between pre- and post-exposure tests, scores in the pre-exposure test were subtracted from scores in the post-exposure test. Positive scores then indicate preferences increased after exposure, whereas negative scores indicate preferences decreased after exposure.

A mixed-model analysis of variance (ANOVA) was carried out with change in preference as the dependent variable, sex of face (male/female) and face trait (sexual dimorphism/symmetry) as within-participant factors, and condition (high pathogen/low pathogen) and sex of participant (male/female) as between-participant factors. This analysis revealed a significant interaction among sex of face, sex of participant and condition (*F*_1,237_ = 13.45, *p* < 0.001, 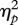 = 0.054). There was also a significant main effect of condition (*F*_1,237_ = 8.36, *p* = 0.004, 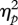 = 0.034), and a significant interaction between sex of participant and sex of face (*F*_1,237_ = 4.02, *p* = 0.046, 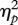 = 0.017), though these were both qualified by the higher-order interaction. No other interactions or main effects were significant (all *F*_1,237_ < 2.29, *p* > 0.116, 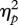 < 0.010).

To parse the three-way interaction, we ran the same ANOVA as above but splitting by sex of participant and removing sex of participant as a factor. For women, this revealed a significant interaction between sex of face and condition (*F*_1,122_ = 8.47, *p* = 0.004, 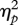 = 0.065). There was a significant effect of condition (*F*_1,122_ = 5.05, *p* = 0.026, 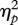 = 0.040) and a close-to-significant effect of sex of face (*F*_1,122_ = 3.73, *p* = 0.056, 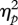 = 0.030), though these were both qualified by the interaction. No other interactions or main effects were significant (all *F*_1,122_ < 1.07, *p* > 0.303, 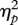 < 0.009). For men, the equivalent analysis also revealed a significant interaction between sex of face and condition (*F*_1,115_ = 5.28, *p* = 0.023, 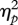 = 0.044). There was a close-to-significant effect of condition (*F*_1,115_ = 3.42, *p* = 0.067, 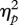 = 0.029). No other interactions or main effects were significant (all *F*_1,115_ < 0.89, *p* > 0.348, 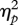 < 0.008).

To further parse the two-way interactions above, we ran separate ANOVAs with trait as the within-subjects variable and condition as the between-subjects variable, split by sex of participant and sex of face. For women, these revealed a significant effect of condition for male faces (*F*_1,122_ = 12.87, *p* < 0.001, 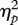 = 0.095), but no significant effect for female faces (*F*_1,122_ = 0.08, *p* = 0.782, 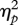 = 0.001). There was no main effect of trait or interaction with trait in either analysis (both *F*_1,122_ < 1.53, *p* > 0.219, 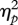 < 0.012). For men, these revealed a significant effect of condition for female faces (*F*_1,115_ = 12.06, *p* < 0.001, 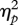 = 0.095), but no significant effects for male faces (*F*_1,115_ = 0.04, *p* = 0.843, 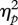 < 0.001). There was no main effect of trait or interaction with trait in either analysis (both *F*_1,115_ < 0.32, *p* > 0.574, 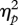 < 0.003).

Although there was no interaction with face trait, we repeated the analyses above separately for each face trait for further clarity. For women, these revealed significant effects of condition for male faces for sexual dimorphism (*F*_1,122_ = 7.99, *p* = 0.005, 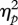 = 0.061) and symmetry (*F*_1,122_ = 5.56, *p* = 0.020, 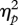 = 0.044), but no significant effects for female faces (sexual dimorphism: *F*_1,122_ = 1.79, *p* = 0.183, 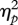 = 0.014; symmetry: *F*_1,122_ = 0.39, *p* = 0.536, 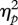 = 0.003). For men, these revealed significant effects of condition for female faces for sexual dimorphism (*F*_1,115_ = 5.68, *p* = 0.019, 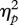 = 0.047) and symmetry (*F*_1,115_ = 6.05, *p* = 0.015, 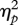 = 0.050), but no significant effects for male faces (sexual dimorphism: *F*_1,115_ = 0.02, *p* = 0.892, 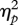 < 0.001; symmetry: *F*_1,115_ = 0.16, *p* = 0.692, 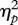 = 0.001).

Finally, to examine whether difference scores differed from chance (0), we split by condition and conducted one-sample *t*-tests. For the pathogen condition, these revealed significant differences for women judging male faces (sexual dimorphism: *t*_67_ = 2.71, *p* = 0.008; symmetry: *t*_67_ = 2.15, *p* = 0.036) but not female faces (sexual dimorphism: *t*_67_ = 1.50, *p* = 0.138; symmetry: *t*_67_ = 0.90, *p* = 0.369), and significant differences for men judging female faces (sexual dimorphism: *t*_55_ = 2.28, *p* = 0.027; symmetry: *t*_55_ = 2.85, *p* = 0.006) but not male faces (sexual dimorphism: *t*_55_ = 0.29, *p* = 0.770; symmetry: *t*_55_ = 0.10, *p* = 0.923). For the neutral condition, no significant differences from chance were found (all *t* < 1.55, all *p* > 0.126).

**Figure 2.**
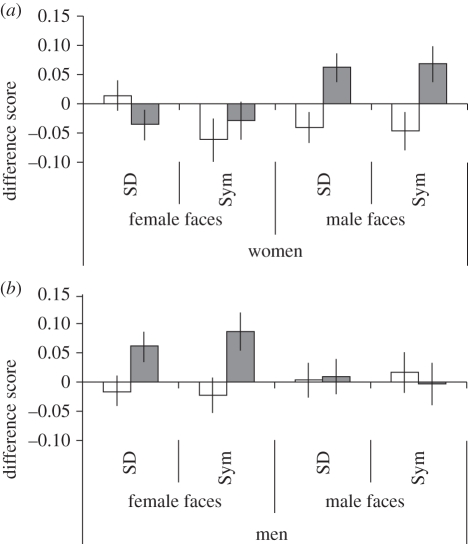
Change in preference (±1 s.e.m.) for symmetry (Sym) and sexual dimorphism (SD) in male and female faces after exposure to pathogen cues (grey bars) or neutral stimuli (white bars) for (*a*) women and (*b*) men.

Together, these analyses demonstrate that preferences for high sexual dimorphism and symmetry are stronger after exposure to cues to environmental pathogens than after exposure to images without these cues. Furthermore, these changes in preferences were restricted to judgements of opposite-sex faces and did not occur for judgements of own-sex faces. This meant that women preferred more masculine and more symmetric male faces and men preferred more feminine and more symmetric female faces after exposure to pathogen cues than when not exposed to such cues. Mean difference scores can be seen in figure 2.

## 4. Discussion

The current study demonstrates that both men and women moderate their preferences for sexually dimorphic facial cues and symmetry according to their recent experience of visual cues to environmental pathogens. Change in preferences was seen only for opposite-sex faces, with women preferring more masculine and more symmetric male faces and men preferring more feminine and more symmetric female faces after exposure to pathogen cues than after not being exposed to such cues. Cues to environmental pathogens had no significant effects on preferences for same-sex faces. Specificity to opposite-sex faces strongly suggests that visual cues to pathogens mediate partner preferences and not preferences for face traits in general. Exposure to visual cues of pathogens increasing preferences for face traits associated with indirect benefits may then be adaptive to increase offspring survival under these conditions. Potentially, such visual exposure may also increase attention to health cues, as being free from infection has a direct benefit in terms of avoiding infection and could also result in a greater ability to provide parental investment in the long term.

Gangestad & Buss [52] have shown that parasite prevalence is positively related to the strength of preference for healthy and attractive partners in men and women (see also [61]). More directly related to face preference, DeBruine *et al*. [54] recently demonstrated that female preferences for male facial masculinity across human populations increased as health factors decreased. Other work has shown that women who reported particularly strong disgust reactions to sources of pathogens also showed particularly strong attraction to male faces with masculine proportions [55]. This previous work was correlational, however, and generally focused exclusively on women's mate preferences. As noted by Brooks *et al.* [56], correlational work is open to additional variables that account for relationships. For example, variation in the extent of inequalities in wealth within countries is also a good predictor of variation for female masculinity preferences across cultures [56]. The data presented in the current study thus provide a useful first step in addressing how experimentally manipulated cues to pathogen prevalence can influence preferences in both men and women. We note that while our study lacks ecological validity, with the slideshow simply being presented, minimal experience was required to influence preferences. Experience with cues to pathogens in the real world may indeed be more effective given that they pose greater threat to individual health and exposure is less likely to be fleeting. Visual experience with pathogens, potentially tied to disgust sensitivity, may be a mechanism that generates both cross-cultural variation in preferences for certain face traits (e.g. [54]) and also individual differences in mate preferences within a culture (e.g. [55]).

As noted earlier, competing predictions can be made concerning environmental harshness in terms of resources or mortality risk. One previous study presented vignettes that suggested a harsh versus a safe environment based on cues to resource availability/scarcity. Imagining oneself as the person in either the high or low resource-availability scenario affected preferences for masculinity in men and women, with a low-resource environment leading to higher preferences for feminine men and masculine women for long-term partnerships [35]. A harsh, low-resource environment then appears to promote a strategy wherein individuals favour low-quality but potentially higher-investing individuals for long-term relationships. This contrasts with the current data, in which cues to pathogens led to higher preferences for quality, possibly at the cost of investment. Potentially, this pattern of data highlights different aspects of environmental influence on preferences. Resource availability and parasite prevalence may drive preferences in different ways.

Previous authors have noted that ecological harshness may favour a low-mating-effort/high-parental-investment strategy [47,48], while others have noted that a harsh environment may lead individuals to maximize their reproductive output by focusing on acquiring good genes for their offspring [49,50]. Our data, combined with previous work, suggest that both arguments have utility and also suggest that mate preferences may be sensitive to the type of environmental harshness in terms of resource availability or parasite prevalence.

In summary, our experiment suggests that exposure to cues of environmental pathogens changes face preferences in both men and women, increasing preferences for proposed good-gene markers in opposite-sex faces. These data complement findings from studies demonstrating individual and cross-cultural differences in mate preferences. Changing preferences according to pathogen cues could generate both variation between cultures and agreement within a culture. As most individuals within a culture will have similar experiences with pathogen cues, this can lead to general within-cultural agreement. As pathogen cues differ consistently between cultures, this can lead to predictable cross-cultural variation, while individual experience may account for some within-culture variability in preference. Our data also suggest that the same person changing environment or with differing experience may alter their preferences. In other words, the link between pathogen prevalence and mate preference is not fixed and inflexible, but can change dynamically according to recent experience. Overall, these data demonstrate that preferences can potentially be strategically flexible according to recent visual experience.

## References

[1] Thornhill R. Gangestad S. W. 1999 Facial attractiveness. Trends Cogn. Sci. 3, 452–460 10.1016/S1364-6613(99)01403-5 (doi:10.1016/S1364-6613(99)01403-5) 10562724

[2] Jasienska G. Lipson S. F. Ellison P. T. Thune I. Ziomkiewicz A. 2006 Symmetrical women have higher potential fertility. Evol. Hum. Behav. 27, 390–400 10.1016/j.evolhumbehav.2006.01.001 (doi:10.1016/j.evolhumbehav.2006.01.001)

[3] Møller A. P. Thornhill R. 1998 Bilateral symmetry and sexual selection: a meta-analysis. Am. Nat. 151, 174–192 10.1086/286110 (doi:10.1086/286110) 18811416

[4] Thornhill R. Gangestad S. W. 2006 Facial sexual dimorphism, developmental stability, and susceptibility to disease in men and women. Evol. Hum. Behav. 27, 131–144 10.1016/j.evolhumbehav.2005.06.001 (doi:10.1016/j.evolhumbehav.2005.06.001)

[5] Weeden J. Sabini J. 2005 Physical attractiveness and health in western societies: a review. Psychol. Bull. 131, 635–653 10.1037/0033-2909.131.5.635 (doi:10.1037/0033-2909.131.5.635) 16187849

[6] Grammer K. Thornhill R. 1994 Human (*Homo sapiens*) facial attractiveness and sexual selection: the role of symmetry and averageness. J. Comp. Psychol. 108, 233–242 10.1037/0735-7036.108.3.233 (doi:10.1037/0735-7036.108.3.233) 7924253

[7] Mealey L. Bridgestock R. Townsend G. 1999 Symmetry and perceived facial attractiveness. J. Personal. Soc. Psychol. 76, 151–158 10.1037/0022-3514.76.1.151 (doi:10.1037/0022-3514.76.1.151) 9972560

[8] Penton-Voak I. S. Jones B. C. Little A. C. Baker S. Tiddeman B. Burt D. M. Perrett D. I. 2001 Symmetry, sexual dimorphism in facial proportions, and male facial attractiveness. Proc. R. Soc. Lond. B 268, 1617–1623 10.1098/rspb.2001.1703 (doi:10.1098/rspb.2001.1703) PMC1088785 11487409

[9] Scheib J. E. Gangestad S. W. Thornhill R. 1999 Facial attractiveness, symmetry, and cues to good genes. Proc. R. Soc. Lond. B 266, 1913–1917 10.1098/rspb.1999.0866 (doi:10.1098/rspb.1999.0866) PMC1690211 10535106

[10] Little A. C. Burt D. M. Penton-Voak I. S. Perrett D. I. 2001 Self-perceived attractiveness influences human female preferences for sexual dimorphism and symmetry in male faces. Proc. R. Soc. Lond. B 268, 39–44 10.1098/rspb.2000.1327 (doi:10.1098/rspb.2000.1327) PMC1087598 12123296

[11] Perrett D. I. Burt D. M. Penton-Voak I. S. Lee K. J. Rowland D. A. Edwards R. 1999 Symmetry and human facial attractiveness. Evol. Hum. Behav. 20, 295–307 10.1016/S1090-5138(99)00014-8 (doi:10.1016/S1090-5138(99)00014-8)

[12] Rhodes G. Proffitt F. Grady J. Sumich A. 1998 Facial symmetry and the perception of beauty. Psychonom. Bull. Rev. 5, 659–669

[13] Little A. C. Jones B. C. 2003 Evidence against perceptual bias views for symmetry preferences in human faces. Proc. R. Soc. Lond. B. 270, 1759–1763 10.1098/rspb.2003.2445 (doi:10.1098/rspb.2003.2445) PMC1691445 12964976

[14] Folstad I. Karter A. J. 1992 Parasites, bright males and the immunocompetence handicap. Am. Nat. 139, 603–622 10.1086/285346 (doi:10.1086/285346)

[15] Rhodes G. Chan J. Zebrowitz L. A. Simmons L. W. 2003 Does sexual dimorphism in human faces signal health? Proc. R. Soc. Lond. B 270, S93–S95 10.1098/rsbl.2003.0023 (doi:10.1098/rsbl.2003.0023) PMC1698019 12952647

[16] Cunningham M. R. Barbee A. P. Pike C. L. 1990 What do women want? Facialmetric assessment of multiple motives in the perception of male facial physical attractiveness. J. Personal. Soc. Psychol. 59, 61–72 10.1037/0022-3514.59.1.61 (doi:10.1037/0022-3514.59.1.61) 2213490

[17] DeBruine L. M. 2006 Correlated preferences for facial masculinity and ideal or actual partner's masculinity. Proc. R. Soc. B 273, 1355–1360 10.1098/rspb.2005.3445 (doi:10.1098/rspb.2005.3445) PMC1560296 16777723

[18] Berry D. S. McArthur L. Z. 1985 Some components and consequences of a babyface. J. Personal. Soc. Psychol. 48, 312–323 10.1037/0022-3514.48.2.312 (doi:10.1037/0022-3514.48.2.312)

[19] Little A. C. Hancock P. J. 2002 The role of masculinity and distinctiveness on the perception of attractiveness in human male faces. Br. J. Psychol. 93, 451–464 10.1348/000712602761381349 (doi:10.1348/000712602761381349) 12519528

[20] Perrett D. I. Lee K. J. Penton-Voak I. S. Rowland D. R. Yoshikawa S. Burt D. M. Henzi S. P. Castles D. L. Akamatsu S. 1998 Effects of sexual dimorphism on facial attractiveness. Nature 394, 884–887 10.1038/29772 (doi:10.1038/29772) 9732869

[21] Little A. C. Jones B. C. Penton-Voak I. S. Burt D. M. Perrett D. I. 2002 Partnership status and the temporal context of relationships influence human female preferences for sexual dimorphism in male face shape. Proc. R. Soc. Lond. B 269, 1095–1100 10.1098/rspb.2002.1984 (doi:10.1098/rspb.2002.1984) PMC1691012 12061950

[22] Thornhill R. Grammer K. 1999 The body and face of woman: one ornament that signals quality? Evol. Hum. Behav. 20, 105–120 10.1016/S1090-5138(98)00044-0 (doi:10.1016/S1090-5138(98)00044-0)

[23] Jasienska G. Ziomkiewicz A. Ellison P. T. Lipson S. F. Thune I. 2004 Large breasts and narrow waists indicate high reproductive potential in women. Proc. R. Soc. Lond. B 271, 1213–1217 10.1098/rspb.2004.2712 (doi:10.1098/rspb.2004.2712) PMC1691716 15306344

[24] Enlow D. M. 1982 Handbook of facial growth, 2nd ed. Philadelphia, PA: Saunders

[25] Law-Smith M. J. 2006 Facial appearance is a cue to oestrogen levels in women. Proc. R. Soc. B 273, 135–140 10.1098/rspb.2005.3296 (doi:10.1098/rspb.2005.3296) PMC1560017 16555779

[26] Cunningham M. R. 1986 Measuring the physical in physical attractiveness: quasi-experiments on the sociobiology of female facial beauty. J. Personal. Soc. Psychol. 50, 925–935 10.1037/0022-3514.50.5.925 (doi:10.1037/0022-3514.50.5.925)

[27] Jones D. Hill K. 1993 Criteria of facial attractiveness in five populations. Hum. Nat. 4, 271–296 10.1007/BF02692202 (doi:10.1007/BF02692202) 24214367

[28] Welling L. L. M. Jones B. C. DeBruine L. M. Smith F. G. Feinberg D. R. Little A. C. Al-Dujaili E. A. S. 2008 Men report stronger attraction to femininity in women's faces when their testosterone levels are high. Horm. Behav. 54, 703–708 10.1016/j.yhbeh.2008.07.012 (doi:10.1016/j.yhbeh.2008.07.012) 18755192

[29] Little A. C. Jones B. C. Waitt C. Tiddeman B. P. Feinberg D. R. Perrett D. I. Apicella C. L. Marlowe F. W. 2008 Symmetry is related to sexual dimorphism in faces: data across culture and species. PLoS ONE 3, e2106 10.1371/journal.pone.0002106 (doi:10.1371/journal.pone.0002106) 18461131 PMC2329856

[30] Little A. C. Jones B. C. DeBruine L. M. Feinberg D. R. 2008 Symmetry and sexual dimorphism in human faces: interrelated preferences suggest both signal quality. Behav. Ecol. 19, 902–908 10.1093/beheco/arn049 (doi:10.1093/beheco/arn049)

[31] Penton-Voak I. S. Little A. C. Jones B. C. Burt D. M. Tiddeman B. P. Perrett D. I. 2003 Female condition influences preferences for sexual dimorphism in faces of male humans (*Homo sapiens*). J. Comp. Psychol. 117, 264–271 10.1037/0735-7036.117.3.264 (doi:10.1037/0735-7036.117.3.264) 14498802

[32] Penton-Voak I. S. Perrett D. I. Castles D. L. Kobayashi T. Burt D. M. Murray L. K. Minamisawa R. 1999 Menstrual cycle alters face preference. Nature 399, 741–742 10.1038/21557 (doi:10.1038/21557) 10391238

[33] Little A. C. Jones B. C. Burt D. M. Perrett D. I. 2007 Preferences for symmetry in faces change across the menstrual cycle. Biol. Psychol. 76, 209–216 10.1016/j.biopsycho.2007.08.003 (doi:10.1016/j.biopsycho.2007.08.003) 17919806

[34] Jones B. C. DeBruine L. M. Perrett D. I. Little A. C. Feinberg D. R. Smith M. J. L. 2008 Effects of menstrual cycle phase on face preferences. Arch. Sexual Behav. 37, 78–84 10.1007/s10508-007-9268-y (doi:10.1007/s10508-007-9268-y) 18193349

[35] Little A. C. Cohen D. L. Jones B. C. Belsky J. 2007 Human preferences for facial masculinity change with relationship type and environmental harshness. Behav. Ecol. Sociobiol. 61, 967–973 10.1007/s00265-006-0325-7 (doi:10.1007/s00265-006-0325-7)

[36] Jones B. C. DeBruine L. M. Little A. C. Conway C. A. Welling L. L. M. Smith F. 2007 Sensation seeking and men's face preferences. Evol. Hum. Behav. 28, 439–446 10.1016/j.evolhumbehav.2007.07.006 (doi:10.1016/j.evolhumbehav.2007.07.006)

[37] Fraccaro P. J. Feinberg D. R. DeBruine L. M. Little A. C. Watkins C. D. Jones B. C. 2010 Correlated male preferences for femininity in female faces and voices. Evol. Psychol. 8, 447–461 10.1177/147470491000800311 PMC10481090 22947812

[38] Little A. C. Apicella C. L. Marlowe F. W. 2007 Preferences for symmetry in human faces in two cultures: data from the UK and the Hadza, an isolated group of hunter–gatherers. Proc. R. Soc. B 274, 3113–3117 10.1098/rspb.2007.0895 (doi:10.1098/rspb.2007.0895) PMC2293939 17925281

[39] Johnston V. S. Hagel R. Franklin M. Fink B. Grammer K. 2001 Male facial attractiveness: evidence for a hormone-mediated adaptive design. Evol. Hum. Behav. 22, 251–267 10.1016/S1090-5138(01)00066-6 (doi:10.1016/S1090-5138(01)00066-6)

[40] Burley N. 1986 Sexual selection for aesthetic traits in species with biparental care. Am. Nat. 127, 415–445 10.1086/284493 (doi:10.1086/284493)

[41] Moller A. P. Thornhill R. 1998 Male parental care, differential parental investment by females and sexual selection. Anim. Behav. 55, 1507–1515 10.1006/anbe.1998.0731 (doi:10.1006/anbe.1998.0731) 9641996

[42] Booth A. Dabbs J. 1993 Testosterone and men's marriages. Social Forces 72, 463–477

[43] Hill K. Hurtado A. M. 1996 Ache life history: the ecology and demography of a foraging people. New York, NY: Aldine de Gruyter

[44] Wilson M. Daly M. 1997 Life expectancy, economic inequality, homicide, and reproductive timing in Chicago neighbourhoods. Br. Med. J. 314, 1271–1274 9154035 10.1136/bmj.314.7089.1271 PMC2126620

[45] Ellison P. T. (ed.) 2001 Reproductive ecology and human evolution. New York, NY: Aldine de Gruyter

[46] Campbell K. L. Wood J. W. (eds) 1994 Human reproductive ecology: interactions of environment, fertility, and behaviour. New York, NY: New York Academy of Sciences

[47] Mace R. 2000 Evolutionary ecology of human life history. Anim. Behav. 59, 1–10 10.1006/anbe.1999.1287 (doi:10.1006/anbe.1999.1287) 10640361

[48] Geary D. C. Vigil J. Byrd-Craven J. 2004 Evolution of human mate choice. J. Sex Res. 41, 27–42 10.1080/00224490409552211 (doi:10.1080/00224490409552211) 15216422

[49] Chisholm J. S. 1996 The evolutionary ecology of attachment organization. Hum. Nat.-Interdiscip. Biosoc. Perspect. 7, 1–37 10.1007/BF02733488 24203250

[50] Belsky J. Steinberg L. Draper P. 1991 Childhood experience, interpersonal development, and reproductive strategy: an evolutionary-theory of socialization. Child Dev. 62, 647–670 10.2307/1131166 (doi:10.2307/1131166) 1935336

[51] Penton-Voak I. S. Jacobson A. Trivers R. 2004 Populational differences in attractiveness judgements of male and female faces: comparing British and Jamaican samples. Evol. Hum. Behav. 25, 355–370 10.1016/j.evolhumbehav.2004.06.002 (doi:10.1016/j.evolhumbehav.2004.06.002)

[52] Gangestad S. W. Buss D. M. 1993 Pathogen prevalence and human mate preferences. Ethol. Sociobiol. 14, 89–96 10.1016/0162-3095(93)90009-7 (doi:10.1016/0162-3095(93)90009-7)

[53] Low B. S. 1990 Marriage systems and pathogen stress in human societies. Am. Zool. 30, 325–339

[54] DeBruine L. M. Jones B. C. Crawford J. R. Welling L. L. M. Little A. C. 2010 The health of a nation predicts their mate preferences: cross-cultural variation in women's preferences for masculinized male faces. Proc. R. Soc. B 277, 2405–2410 10.1098/rspb.2009.2184 (doi:10.1098/rspb.2009.2184) PMC2894896 20236978

[55] DeBruine L. M. Jones B. C. Tybur J. M. Lieberman D. Griskevicius V. 2010 Women's preferences for masculinity in male faces are predicted by pathogen disgust, but not by moral or sexual disgust. Evol. Hum. Behav. 31, 69–74 10.1016/j.evolhumbehav.2009.09.003 (doi:10.1016/j.evolhumbehav.2009.09.003)

[56] Brooks R. Scott I. Maklakov A. A. Kasumovic M. M. Clark A. P. Penton-Voak I. S. 2010 National income inequality predicts women's preferences for masculinised faces better than health does. Proc. R. Soc. B 278, 810–812 10.1098/rspb.2010.0964 (doi:10.1098/rspb.2010.0964) PMC3049041 21147809

[57] Jones B. C. 2005 Menstrual cycle, pregnancy and oral contraceptive use alter attraction to apparent health in faces. Proc. R. Soc. B 272, 347–354 10.1098/rspb.2004.2962 (doi:10.1098/rspb.2004.2962) PMC1634990 15734688

[58] Benson P. J. Perrett D. I. 1993 Extracting prototypical facial images from exemplars. Perception 22, 257–262 10.1068/p220257 (doi:10.1068/p220257) 8316513

[59] Tiddeman B. P. Burt D. M. Perrett D. I. 2001 Prototyping and transforming facial texture for perception research. IEEE Comp. Graph. Appl. 21, 42–50 10.1109/38.946630 (doi:10.1109/38.946630)

[60] Curtis V. Aunger R. Rabie T. 2004 Evidence that disgust evolved to protect from risk of disease. Proc. R. Soc. Lond. B 271, S131–S133 10.1098/rsbl.2003.0144 (doi:10.1098/rsbl.2003.0144) PMC1810028 15252963

[61] Gangestad S. W. Haselton M. G. Buss D. M. 2006 Evolutionary foundations of cultural variation: evoked culture and mate preferences. Psychol. Inquiry 17, 75–95 10.1207/s15327965pli1702_1 (doi:10.1207/s15327965pli1702_1)

